# The glucose lowering effects of CL 316,243 dissipate with repeated use and are rescued bycilostamide

**DOI:** 10.14814/phy2.15187

**Published:** 2022-02-18

**Authors:** Kyle D. Medak, Greg L. McKie, Hesham Shamshoum, Ian Seguin, David C. Wright

**Affiliations:** ^1^ Department of Human Health and Nutritional Sciences University of Guelph Guelph Ontario Canada

**Keywords:** adipose tissue, cilostamide, CL 316,243, fatty acids, insulin, mitochondria, thermogenesis, obesity, UCP1

## Abstract

Repeated activation of the beta 3 adrenergic receptor (β3AR) with the agonist CL 316,243 (CL) results in remodeling of white adipose tissue (WAT) characterized by increased mitochondrial enzymes and expression of uncoupling protein 1 (UCP1). β3AR activation also has profound acute metabolic effects including rapidly decreasing blood glucose, secondary to fatty acid‐induced increases in insulin, and increasing energy expenditure. The acute (single dose) effects of β3AR activation have largely been examined in treatment naive animals and under room temperature housing conditions. The current study examined if repeated CL treatment would lead to an attenuation of acute metabolic effects of CL treatment under thermoneutral housing conditions and if this could be rescued with cilostamide, a phosphodiesterase inhibitor. We provide evidence demonstrating that the acute effects of CL to increase serum fatty acids and insulin and reduce blood glucose, but not increases in energy expenditure, are attenuated in mice following repeated treatment with CL. This occurs in parallel with reductions in indices of protein kinase A signaling in WAT including the phosphorylation of hormone sensitive lipase. The findings of attenuated serum fatty acid, insulin, and blood glucose responses were confirmed in both high‐fat fed and UCP1^−/−^ mice repeatedly treated with CL. Desensitization to CL in mice was rescued by cilostamide. Herein, we provide evidence that the glucose lowering, but not thermogenesis inducing, effects of CL are attenuated with repeated treatment and can be rescued by cilostamide. The findings of this study point toward novel adjunct treatment approaches that could be used to maximize therapeutic, glucose lowering effects of β3AR agonists.

## Graphical abstract

We demonstrate that repeated treatment with CL 316,243 leads to a blunted ability of the drug to acutely reduce blood glucose. This effect is seen in several models including high fat fed and UCP1^−/−^ mice and can be rescued by cotreatment with the phosphodiesterase inhibitor, cilostamide. The findings of this paper point towards adjunct treatment approaches that could be used to maximize the glucose lowering effects of CL 316,243.

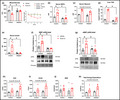

## 1 INTRODUCTION

Beta 3 adrenergic receptors (β3ARs) are highly expressed in white and brown adipose tissue and the pharmacological targeting of this receptor results in beneficial metabolic effects in both rodents (Bloom et al., 1992) and humans (Weyer et al., 1998). Repeated dosing with CL 316,243 (CL) results in a marked remodeling of white adipose tissue characterized by increases in the content of mitochondrial enzymes, the expression of uncoupling protein 1 (UCP1) and the appearance of multilocular fat cells (Buzelle et al., 2015; Mottillo et al., 2014, 2016). These hallmark features of “browning” occur most prominently in inguinal subcutaneous adipose tissue following CL treatment and are paralleled by reductions in adiposity and increases in energy expenditure (Buzelle et al., 2015; Mottillo et al., 2014). From a therapeutic standpoint the effects of CL are maintained in obesity with high‐fat fed mice displaying decreases in body mass and adiposity and increases in energy expenditure following repeated CL treatment (Xiao et al., 2015).

In addition to the chronic effects of repeated treatment with CL, β3AR agonists lead to rapid and robust alterations in systemic fuel metabolism including the activation of brown adipose non‐shivering thermogenesis (Mottillo et al., 2016) and increases in whole‐body energy expenditure (Gavrilova et al., 2000). At least in mice housed at thermal neutrality (~30°C) (Crane et al., 2014), but perhaps not at room temperature (Granneman et al., 2003; Mottillo et al., 2016), CL‐induced increases in energy expenditure are dependent upon the activation of uncoupling protein 1 (UCP1). The activation of β3ARs also results in a rapid lowering of blood glucose (MacPherson et al., 2014) that is thought to be secondary to fatty acid‐induced increases in insulin secretion (Yoshida et al., 1994). In support of this the attenuation of fatty acid release by injection of nicotinic acid or the knockout of lipolytic enzymes such as ATGL (adipose tissue triglyceride lipase), attenuates CL‐induced increases in circulating insulin and reductions in blood glucose in mice (MacPherson et al., 2014). It is thought that CL‐induced increases in insulin secretion plays an important role in the provision of substrates to activated brown adipose tissue (Heine et al., 2018).

While the potential mechanisms mediating the glucose lowering effects of CL have been well studied, these investigations have been conducted in lean, healthy mice and it is yet to be determined if similar effects would be seen in conditions of preexisting metabolic dysfunction. This is not a trivial matter as CL‐induced increases in indices of lipolysis in vivo are blunted in mice that have been made obese by the provision of a high‐fat diet (Mowers et al., 2013) and studies comparing high‐fat diet to control have found attenuated effects of CL under high‐fat diet conditions (Clookey et al., 2019), thus it is not clear if the glucose lowering effects of CL would remain intact following adaptation to CL in this model.

A further consideration when studying the acute metabolic effects of CL is that these investigations have, for the most part, been completed in drug naive animals. This is an important point as prior work has shown that chronic cold stress, an intervention that activates β3ARs, results in a desensitization of brown adipocytes to beta adrenergic stimulation (Unelius et al., 1993), a finding consistent with data reporting decreases in lipolytic responsiveness in adipocytes from rats treated with CL for 7 days (Atgié et al., 1998) and reductions in acute β3AR agonist‐induced increases in serum glycerol from rats repeatedly dosed with a β3AR agonist (Mills, 2000). From a mechanistic perspective, reductions in beta adrenergic responsiveness have been paralleled by increases in phosphodiesterase activity (Unelius et al., 1993), an enzyme which degrades cAMP, an effector of beta‐adrenergic activation. Given these findings, it is not clear if the robust glucose lowering effects of acute activation of β3ARs dissipates with repeated treatment and if so, if the pharmacological targeting of phosphodiesterase would be sufficient to overcome this.

The purpose of the current investigation was to determine if the acute metabolic effects of CL, including increases in insulin and reductions in blood glucose would dissipate with repeated use in both lean and obese mice. As it has been suggested that CL‐induced increases in insulin serves as a mechanism to provide substrate to activate brown adipose tissue (Heine et al., 2018), we further wanted to determine if the ablation of UCP1, a model of reduced brown adipose activation, would alter the acute effects of CL, thus providing further insight into the relationship between CL‐induced increases in insulin and brown adipose tissue. Finally, we wanted to determine if co‐treatment with a phosphodiesterase inhibitor, cilostamide, would rescue the acute effects of CL on glucose and insulin in mice repeatedly treated with CL. We hypothesized that repeated treatment with CL would lead to an attenuation of the acute glucose lowering effects of this compound, that this would be maintained in both high‐fat fed and UCP1^−/−^ mice, and that co‐treatment with a phosphodiesterase inhibitor would potentiate this effect.

## 2 MATERIALS AND METHODS

### 2.1 Animals

All experimental procedures were approved by the University of Guelph Animal Care Committee and followed Canadian Council on Animal Care guidelines. C57BL/6J male mice of 8–12 week old were purchased from Jackson Laboratories. UCP1^−/−^ (B6.129‐*Ucp1^tm1Kz^*/J) breeding pairs were purchased from Jackson Laboratories and bred in house. Mice were group housed in clear polycarbonate shoebox‐style cages (dimensions: 7 1⁄2” × 11 1⁄2” × 5”) with wire lids. Rooms were maintained at ~29°C on a 12:12 h light dark cycle. Animals were given free access to water and standard rodent chow (7004‐Teklad S‐2335 Mouse Breeder Sterilizable Diet; Teklad Diets Harlan Laboratories) or a high‐fat diet (45% kcal from fat; Diet Formula: D12451; Research Diets Inc.).

### 2.2 Materials

Injections were carried out using 25‐gauge needles purchased from ThermoFisher Scientific (cat. no. BD B305122). Dimethylsulfoxide (DMSO) was from Wako Pure Chemical Industries (cat. no. 67‐68‐5). Kolliphor EL was from Millipore Sigma (cat. no. C5135). Primary antibodies against Hormone Sensitive Lipase (HSL; cat. no. 4107), phospho‐HSL Serine660 (cat. no. 4126), and cytochrome *c* (cat. no. 4272) were purchased from Cell Signaling. Primary antibodies against citrate synthetase (CS; cat. no. 129095), pyruvate dehydrogenase E1‐α (PDH; cat. no. 110330), ubiquinol‐cytochrome *c* reductase (CORE1; cat. no. 110252), cytochrome c oxidase subunit 4 (COX IV; cat. no. 16056), uncoupling protein 1 (UCP1; cat. no. 10983) were purchased from Abcam. Antibodies against Vinculin (cat. no. 05386) were purchased from Millipore. Ponceau S (Sigma Aldrich) staining was used as a loading control for immunoblotting (Romero‐Calvo et al., 2010). Secondary antibodies (donkey anti‐rabbit: cat no. 711‐165‐122; and goat anti‐mouse: cat no. 115‐005‐003; IgG) were purchased from Jackson ImmunoResearch. Reagents for SDS‐PAGE, including nitrocellulose membranes and enhanced chemiluminescence, were purchased from Bio‐Rad. Molecular weight marker was purchased from FroggaBio. All additional chemicals, including those used to homogenize samples, were purchased from Sigma‐Aldrich. ELISAs obtained from Mercodia Inc. (Winston‐Salem, NC 27103, USA) were used to measure serum insulin (cat. no. 10‐1247‐01). Serum nonesterified fatty acid (NEFA) (Wako Bioproducts) and glycerol (F6428; Millipore Sigma) were measured on 96‐well plates as previously described (MacPherson et al., 2014).

### 2.3 CL 316,243 treatment

Mice were “acclimated” to CL for 6 days with daily injections of a weight adjusted bolus (1 mg/kg body weight I.P.) or an equivalent volume of saline during their light cycle. Following the 6‐day acclimation to CL, mice were treated acutely with CL (1 mg/kg body weight I.P.) or an equivalent volume of saline to give the following groups: “control‐vehicle”, “control‐acute CL”, “adapted‐vehicle”, “adapted‐acute CL”. Following this acute treatment, blood glucose was measured in the fed state using a handheld glucometer sampled from a drop of blood taken from the tail vein using a single distal tail snip. At 1 or 4 h after acute CL treatment, mice were anesthetized with sodium pentobarbital (5 mg/100 g body weight I.P.) and liver, inguinal white adipose tissue (iWAT; a subcutaneous fat depot), epididymal white adipose tissue (eWAT; an abdominal fat depot), and interscapular brown adipose tissue (BAT) were dissected, weighed, and snap frozen in liquid nitrogen. Cardiac blood was collected via cardiac puncture with 25‐gauge needles and allowed to clot for ~20 min at room temperature then centrifuged at 5000*g* for 10 min at 4°C. Serum was collected and stored at −80°C until further analysis.

### 2.4 Cilostamide treatment

Mice were acclimated to 6 days of CL treatment (1 mg/kg body weight I.P.) then treated with acute CL (1 mg/kg body weight I.P.) and/or cilostamide (10 mg/kg body weight I.P.) or vehicle solution (Figure 5g) to give the following groups: “saline‐vehicle”, “CL‐vehicle”, “saline‐cilostamide”, “CL‐cilostamide”; all of which had been adapted to 6 days of CL. This cilostamide dose has been used previously in mice to inhibit phosphodiesterase 3b measured in WAT (Guirguis et al., 2013). Cilostamide was prepared in 10% DMSO, 5% kolliphor. At 1 h following this acute treatment, blood glucose measurement, and tissue collection were carried out as described above.

### 2.5 Comprehensive laboratory animal monitoring system

Mice were sedated with sodium pentobarbital (~3 mg/100 g body weight) and then subsequently treated with vehicle or CL before being placed into the Comprehensive laboratory animal monitoring system (CLAMS) metabolic caging for the following hour, during which VCO_2_, VO_2_, respiratory exchange ratio (RER; VCO_2_/VO_2_), and energy expenditure were measured. CLAMS caging experiments occurred at ~9:00 am during the animal's light cycle.

### 2.6 White adipose tissue explants

To assess lipolysis ex vivo, adipose tissue explants were prepared immediately after terminal surgeries in drug naive mice or mice acclimated to 6 days of CL (1 mg/kg body weight I.P.). Following anesthetization with sodium pentobarbital (5 mg/100 g body weight I.P.), white adipose tissue was harvested and ~20–25 mg was incubated in 250 µl of M199 supplemented with 2% BSA and left to equilibrate for 30 min in a cell incubator at 37°C and 95% CO_2_/5% O_2_. Following 30 min, cultures were treated with sterile water (control) or 10 μM CL 316,243 for another 2 h. At the end of the experiment, 200 µL of media was collected and stored at −80°C for further analyses. Tissue mass was recorded to normalize NEFA and glycerol accumulation per milligram of adipose tissue.

### 2.7 Liver TAGs

To quantify liver TAG content, snap frozen liver was chipped into ~30 mg pieces, homogenized in 1 ml of 1:2 methanol:chloroform, and agitated overnight at 4°C (Townsend et al., 2019). One ml of 4 mM MgCl was added the following day, vortexed, and centrifuged for 1 h at 1000*g* at 4°C. The organic infranatant was extracted, evaporated overnight in a fume hood, and reconstituted in a 3:2 butanol‐Triton X‐114 mix. TAG content was measured with a commercially available kit (Sigma‐Aldrich, cat. no.F6428) in duplicate.

### 2.8 Immunoblotting

Liver and inguinal adipose were homogenized, protein extracted, quantified, and immunoblotting was completed as we have described in detail previously (Peppler et al., 2018; Snook et al., 2016). In short, membranes were incubated overnight at 4°C with gentle rocking in antibodies diluted (1:1000) with TBST and 5% BSA. The following morning membranes were briefly washed in TBST and incubated for 1 h at room temperature with horseradish peroxidase‐conjugated secondary antibodies (1:2000). Proteins of interest were expressed relative to an internal ponceau loading control or vinculin from the same respective gel as the protein of interest. Please note that a ponceau stain was used for correction purposes for each protein of interest but for space purposes only one representative ponceau is shown for each tissue.

### 2.9 Real time PCR

WAT (100–130 mg) was homogenized in 1 ml of QIAzol (cat. no. 15596018; ThermoFisher Scientific) in a bead mill followed by RNA extraction using a RNeasy mini kit as per the manufacturer's instructions (cat. no. 74104; Qiagen), including DNase free treatment with a commercially available kit (Cat # AM1906; ThermoFisher Scientific) as per Peppler et al. (2019). Synthesis of cDNA was completed using SuperScript II (Cat # 18064014; ThermoFisher Scientific). PCR was run with Sso Advanced Universal SYBR Green Supermix (BioRadCAT#1725271) using PCR primers listed in Table S1, (https://figshare.com/s/11f273becba25a7b38a6) on a Bio‐Rad CFX connect system. All markers are expressed relative to Ppib, which did not change between groups. Relative differences in mRNA expression were determined using the 2^−∆∆CT^ method and normalized to the respective control group.

### 2.10 Statistical analyses

Statistical tests were completed using GraphPad Prism v.9.0 (GraphPad Software). Differences between two groups were determined using an unpaired, two‐tailed *t*‐test. The effects of drug treatments were analyzed by two‐way ANOVA (6‐day CL acclimation or saline × acute CL or saline), followed by Tukey's post hoc analysis if there was a significant interaction between treatments. Significant main effects are indicated by bars above the graph. If the ANOVA indicated a significant interaction then pair‐wise comparisons are displayed. Data sets were analyzed for outliers with Grubbs’ test using GraphPad Outlier Calculator and values were excluded if identified as outliers. Glucose curves are displayed but were not statistically analyzed as this information was represented in AUC values. Normality was assessed using the Shapiro–Wilk test unless a sample size was large enough to use the D’Agostino & Pearson test, as per the recommendation of GraphPad Statistics Guide. If normality tests failed, data were logarithmically transformed (log_10_) to ensure equal variance and normal distribution. A relationship was considered significant when *p* < 0.05.

## 3 RESULTS

### 3.1 6‐days of CL treatment causes weight loss and increases WAT mitochondrial proteins

**FIGURE 1:**
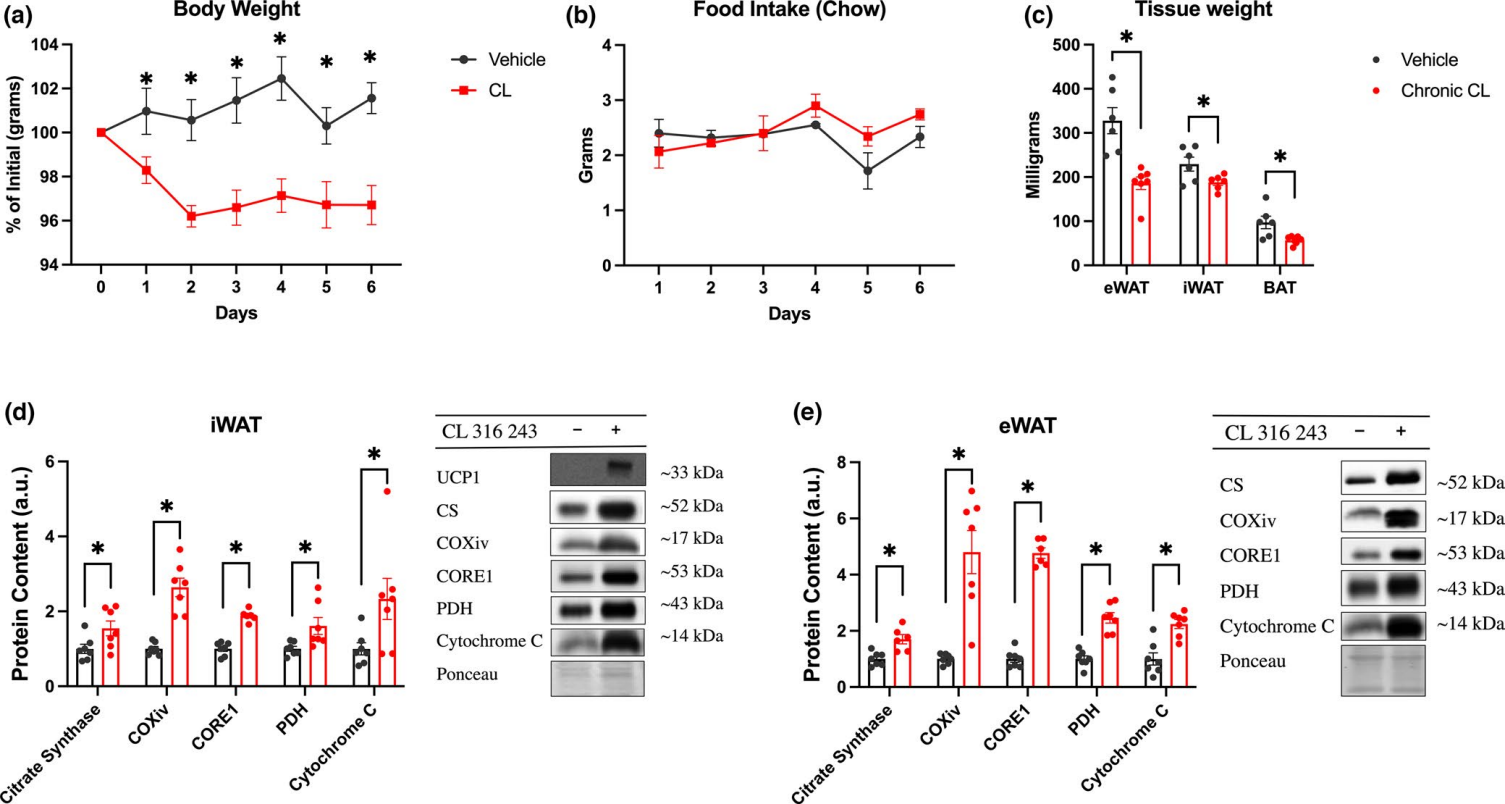
Adaptation to 6‐day CL treatment induces weight loss and increased WAT mitochondrial proteins. Mice were treated with I.P. injections of CL (1 mg/kg) for 6 days. (a, b) Body weight and food intake measured daily throughout treatment (*n* = 6–7 mice/group). Twenty‐four hours following the last CL treatment adipose tissue depots were dissected, weighed (c; *n* = 6–7 mice/group), and analyzed for protein content via western Blot (d, e; *n* = 5–6 mice/group). Proteins of interest were expressed relative to an internal ponceau loading control from the same respective gel as the protein of interest. Please note that a ponceau stain was used for correction purposes for each protein of interest but for space purposes only one representative ponceau is shown for each tissue. Differences between groups were analyzed by two‐tailed unpaired *t*‐test. *p < 0.05 between indicated groups (vehicle vs chronic CL). All data are presented as mean ± SEM

To characterize the effects of CL treatment (1 mg/kg I.P. per day) mice were dosed with a weight matched bolus of CL for 6 days with body weight and food intake monitored daily. Body weight (Figure 1a), represented as a percentage of initial weight, was significantly lower in CL‐treated mice from day 1 post‐treatment and throughout the experiment. Food intake was not significantly changed between treatment groups on any day of treatment (Figure 1b). Twenty‐four‐hours after the last treatment, adipose depots were dissected and weighed, revealing a significant reduction in adipose weight with CL (Figure 1c). Consistent with what we and others have previously reported (Buzelle et al., 2015; Mottillo et al., 2014), CL treatment increased the content of multiple mitochondrial proteins in both iWAT (Figure 1d) and eWAT (Figure 1e) adipose tissue depots. UCP1 protein was undetectable in iWAT from saline treated mice but was robustly increased with CL treatment (Figure 1d). As we could not detect UCP1 in vehicle‐treated mice quantified data are not presented. Similar to previous work (Mottillo et al., 2014), UCP1 protein was not detected in eWAT (data not shown). Collectively our data demonstrates the expected effects of CL on body mass and the induction of mitochondrial proteins in adipose tissue from mice housed at thermoneutrality.

### 3.2 The acute glucose lowering effects of CL dissipate with repeated use

**FIGURE 2:**
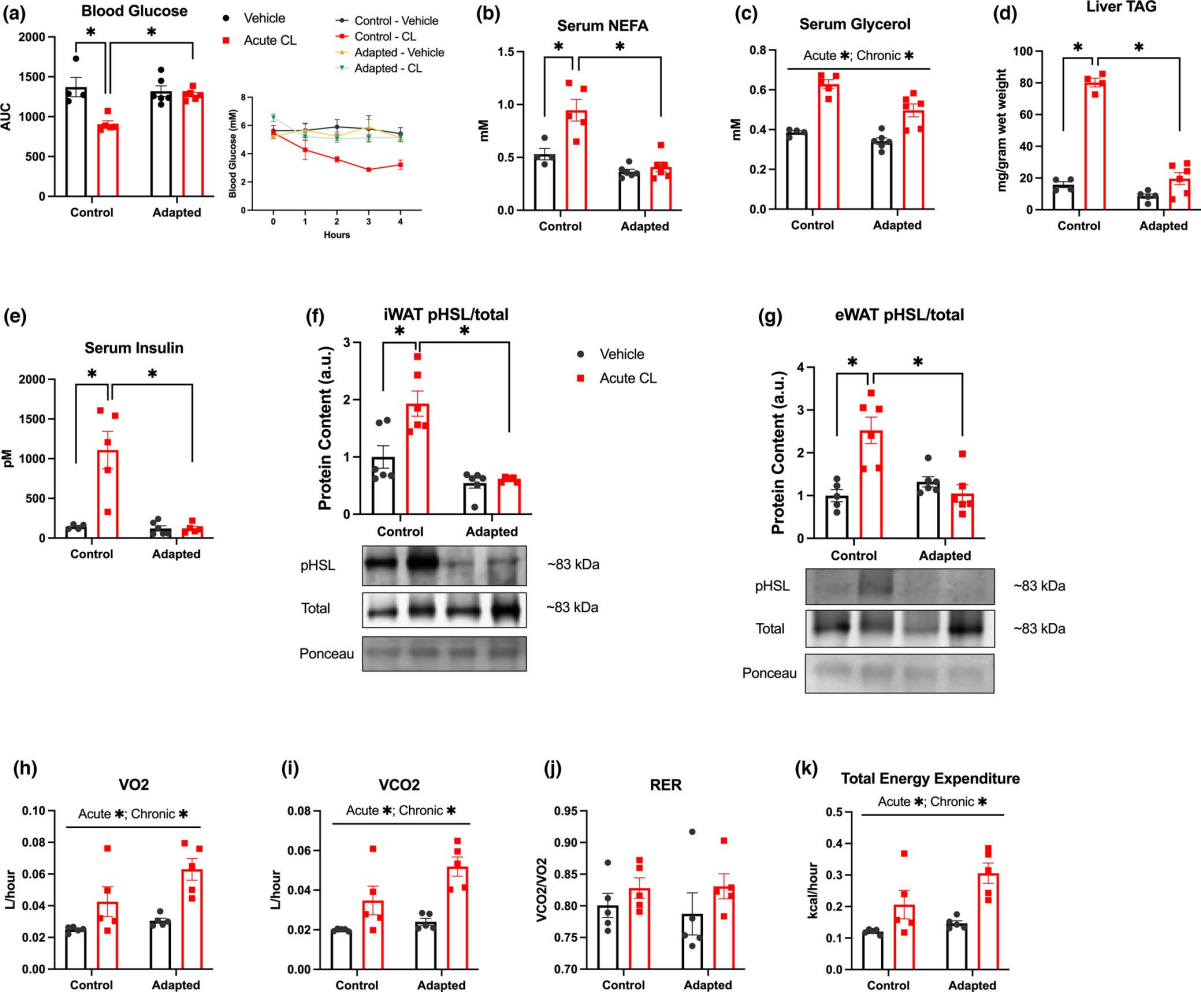
The acute metabolic effects of CL dissipate with repeated use but whole‐body substrate oxidation is intact. Mice (*n* = 4–6 mice/group) were adapted to CL or saline injections for 6 days then treated acutely with CL or saline. (a) Blood glucose was measured each hour for 4 h for determination of AUC (glucose curve inset). Four hours following acute treatment serum, liver, and WAT depots were collected for analysis of serum NEFA (b), glycerol (c), liver TAG accumulation (d), and insulin (e). Mice‐ treated chronically and/or acutely with CL as described above were sacrificed 1 h following the acute treatment for determination of WAT HSL phosphorylation (serine 660) via western blot (f, g). Proteins of interest were expressed relative to an internal ponceau loading control from the same respective gel as the protein of interest. Please note that a ponceau stain was used for correction purposes for each protein of interest but for space purposes only one representative ponceau is shown for each tissue. Phosphorylation of proteins are represented relative to their respective total protein content and ponceau stain loading control (representative images inset). (h, k) Mice were treated chronically with CL or saline for 6 days then anesthetized with sodium pentobarbitol and treated acutely with CL or saline, then placed in the CLAMS system for 1 h for determination of VO_2_, VCO_2_, RER, and total energy expenditure. Differences between groups were analyzed by two‐way ANOVA. *p < 0.05 between indicated groups. All data are presented as mean ± SEM

Having replicated previous findings (Buzelle et al., 2015; Mottillo et al., 2014), on the adaptations with repeated CL treatment, we next aimed to compare the acute effects of CL in animals that are adapted or naive to the drug. To accomplish this, we treated mice for 6 days with CL (1 mg/kg) or saline. On the seventh day half of the animals of each group were treated with acute CL (1 mg/kg) or saline. Blood glucose was measured in vivo each hour for 4 h and revealed a reduction in blood glucose AUC in the drug naive control animals which received acute CL (Figure 2a), an effect that was absent in CL‐adapted mice. As prior work has reported sex differences in the response to CL (Kim et al., 2016) we repeated our experiments in female mice. When examining the glucose AUC in naïve and adapted mice following a single treatment with CL there was a significant interaction such that CL decreased the blood glucose AUC in both naïve (vehicle: 1374 ± 41, acute CL: 822 ± 32, *N* = 5–6/group *p* < 0.05) and adapted (vehicle: 1383 ± 28, acute CL: 1189 ± 28, *N* = 6/group *p* < 0.05) mice, with the glucose AUC being greater (*p* < 0.05) in mice having undergone repeated treatment with CL prior to the acute challenge.

We have previously provided evidence that the glucose lowering effects of CL are mediated by fatty acid‐induced increases in serum insulin (MacPherson et al., 2014). In support of this an attenuation of fatty acid release by injection of nicotinic acid or the knockout of ATGL blunts CL‐induced increases in circulating insulin and reductions in blood glucose. Consistent with this idea, serum NEFA measured 4 h after treatment was increased in the control‐acute CL group but not in adapted animals (Figure 2b). Likewise, there were main effects on serum glycerol to be increased by acute CL and reduced with adaptation to the drug (Figure 2c). Consistent with increase in indices of lipolysis, and presumably the delivery of fatty acids to the liver, acute CL treatment in drug naïve mice led to increases in liver TAG accumulation which was blunted in mice adapted to CL (Figure 2d). In line with a working model of CL‐induced reductions in blood glucose being dependent upon fatty acid mediated increases in serum insulin, acute CL‐induced increases in insulin were absent in CL‐adapted mice (Figure 2e).

To gain insight into the potential mechanisms that could be mediating reductions in adipose tissue lipolysis we assessed the phosphorylation status of HSL on serine 660, a PKA phosphorylation site (Krintel et al., 2009), in tissue taken 1 h after acute treatment. Analysis of serum parameters (NEFA, glycerol, insulin) at this time point yielded the same results as measured at 4 h post‐treatment (Table S2, https://figshare.com/s/9c007f044b77c2d64c31). As shown in Figure 2 acute CL‐induced increases in HSL phosphorylation in both iWAT (Figure 2f) and eWAT (Figure 2g) were absent in mice repeatedly treated with CL. Similar to the blunted increase in HSL phosphorylation, the ability of CL to induce UCP1 and PGC1α mRNA, which are PKA target genes (MacPherson et al., 2016), was absent in eWAT and iWAT from mice repeatedly dosed with CL (Figure S1, https://figshare.com/s/6e39c2de414391feebeb).

To determine if CL had direct effects on adipose tissue lipolysis after repeated treatments, we dosed mice with saline or CL for 6 days and then treated adipose tissue explants from these animals with either saline or CL for 2 h. NEFA accumulation in the culture media from iWAT explants was increased in the control‐acute CL group and not the adapted‐acute CL group (Table 1). Similarly, glycerol accumulation measured in the same iWAT culture media had main effects of acute CL to increase glycerol but a main effect of CL adaptation to decrease glycerol in the media. In eWAT explants, NEFA and glycerol accumulation in culture media was increased by acute CL in animals that were adapted to chronic CL and treated with saline, but the acute increase was significantly greater in explants from naïve mice (Table 1).

**TABLE 1:** White adipose tissue explant metabolites

	Treatment groups				*p* Value		
	Control‐vehicle	Control‐CL	Adapted‐vehicle	Adapted‐CL	Acute CL	Chronic	Acute × chronic
iWAT NEFA (mM/g tissue)	0.014 ± 0.0015^a^	0.027 ± 0.0034^b^	0.013 ± 0.0005^a^	0.015 ± 0.0010^a^	0.0015	0.0042	0.0124
iWAT GLYCEROL (mM/g tissue)	0.024 ± 0.0018	0.040 ± 0.0045	0.019 ± 0.0011	0.030 ± 0.0008	<0.0001	0.0111	0.2595
eWAT NEFA (mM/g tissue)	0.012 ± 0.0009^a^	0.037 ± 0.0017^b^	0.013 ± 0.0011^a^	0.021 ± 0.0016^c^	<0.0001	<0.0001	<0.0001
eWAT GLYCEROL (mM/g tissue)	0.022 ± 0.0014^a^	0.050 ± 0.0030^b^	0.022 ± 0.0021^a^	0.038 ± 0.0025^c^	<0.0001	0.0179	0.0199

Note

Mice were adapted to CL with daily I.P. injection for 6 days in vivo before animals were anesthetized, WAT was dissected, and treated acutely in vitro. Data are represented in mM per hour, per gram of adipose as the mean ± SEM. eWAT, epididymal white adipose tissue; iWAT, inguinal white adipose tissue; NEFA, nonesterified fatty acids; Acute CL, *p* value for main effect of acute CL 316,243 treatment; Chronic, *p* value for main effect of adaptation to 6 days of CL 316,243 treatment; acute × chronic, *p* value for interaction between acute CL treatment and adaptation to chronic CL treatment. Different superscript lowercase letters denote a significant post hoc comparison (*n* = 5 per group).

In contrast to our data demonstrating a blunted glucose lowering effect of CL in adapted mice, there were main effects of acute CL treatment and adaptation to repeated CL to increase VO_2_ (Figure 2h), VCO_2_ (Figure 2i), and total energy expenditure (Figure 2k), with no differences in RER (Figure 2j). Collectively our findings demonstrate that prior adaptation to CL treatment leads to a desensitized glucoregulatory response upon acute CL challenge whereas the ability of CL to acutely increase energy expenditure is unaffected by prior treatments.

### 3.3 The acute metabolic effects of CL dissipate with repeated use in high fat diet‐fed animals

**FIGURE 3:**
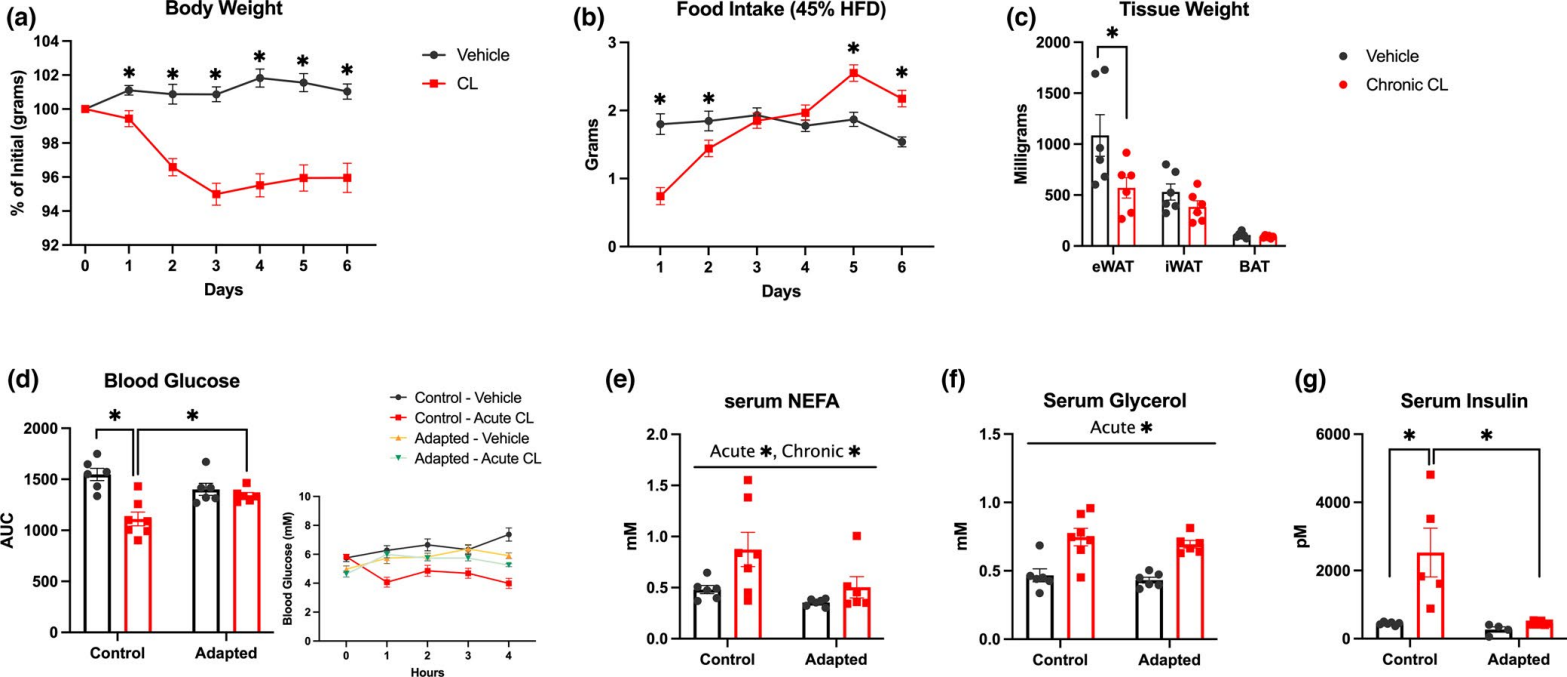
The acute metabolic effects of CL dissipate with repeated use in high fat diet‐fed animals. All mice in these sets of experiments were fed a 45% kcal high‐fat diet for 4 weeks prior to drug treatment then adapted to CL or saline injections for 6 days. On the seventh day mice were treated acutely with CL or saline. (a, b) Body weight and food intake were measured daily throughout treatment (*n* = 12–13 mice/group). (c; *n* = 6 mice/group) Four hours following the acute CL treatment adipose tissue depots were dissected and weighed. (d; *n* = 6–7 mice/group) Blood glucose was measured each hour for 4 h for determination of AUC (glucose curve inset). Four hours following acute treatment serum and WAT depots were collected for analysis of NEFA (e; *n* = 6–7 mice/group), glycerol (f; *n* = 6–7 mice/group), and insulin (g; *n* = 4–6 mice/group). Differences between groups were analyzed by two‐way ANOVA. *p < 0.05 between indicated groups. All data are presented as mean ± SEM

Given the marked effects of CL to reduce blood glucose in drug naïve mice we wanted to determine if this effect was maintained in obese mice, and if a similar adaptation to repeated treatment, as seen in chow fed animals, occurred. Our study design began with 4 weeks of high‐fat diet feeding (45% kcal from fat) before the start of our drug treatment protocol. Mice fed a high‐fat diet were heavier than chow fed animals used in our first set of experiments (high fat: 28.64 ± 0.51 g, chow: 23.18 ± 0.27 g; *p* > 0.0001). During the 6‐day treatment with CL we tracked body weight and food intake. The percent of initial body weight was significantly lower in CL‐treated mice from day 1 post‐treatment and throughout the experiment (Figure 3a). Food intake of the CL‐treated group was significantly lower for 2 days from the beginning of treatment, not different on day 3 to 4, then significantly higher for the final 2 days of the treatment protocol (Figure 3b). In animals treated with CL for 6 days, weight of eWAT but no other fat depots were significantly reduced (Figure 3c). Blood glucose AUC was lower in drug naïve animals after the 4‐h CL treatment compared to control‐vehicle and adapted‐acute CL groups (Figure 3d). Serum NEFA measured 4 h after acute treatment was increased by acute CL and reduced by adaptation to repeated CL (main effect) while serum glycerol was increased by acute CL (Figure 3e,f). Serum insulin was increased in the control‐acute CL group and not in the adapted‐acute CL group (Figure 3g). Collectively, these findings effectively replicate what was shown in lean animals suggesting that nutritional state does not influence this change in responsiveness to repeated CL treatments.

### 3.4 In UCP1 knockout mice the chronic effects of CL to increase mitochondrial proteins are intact and the acute metabolic effects of CL dissipate with repeated use

**FIGURE 4:**
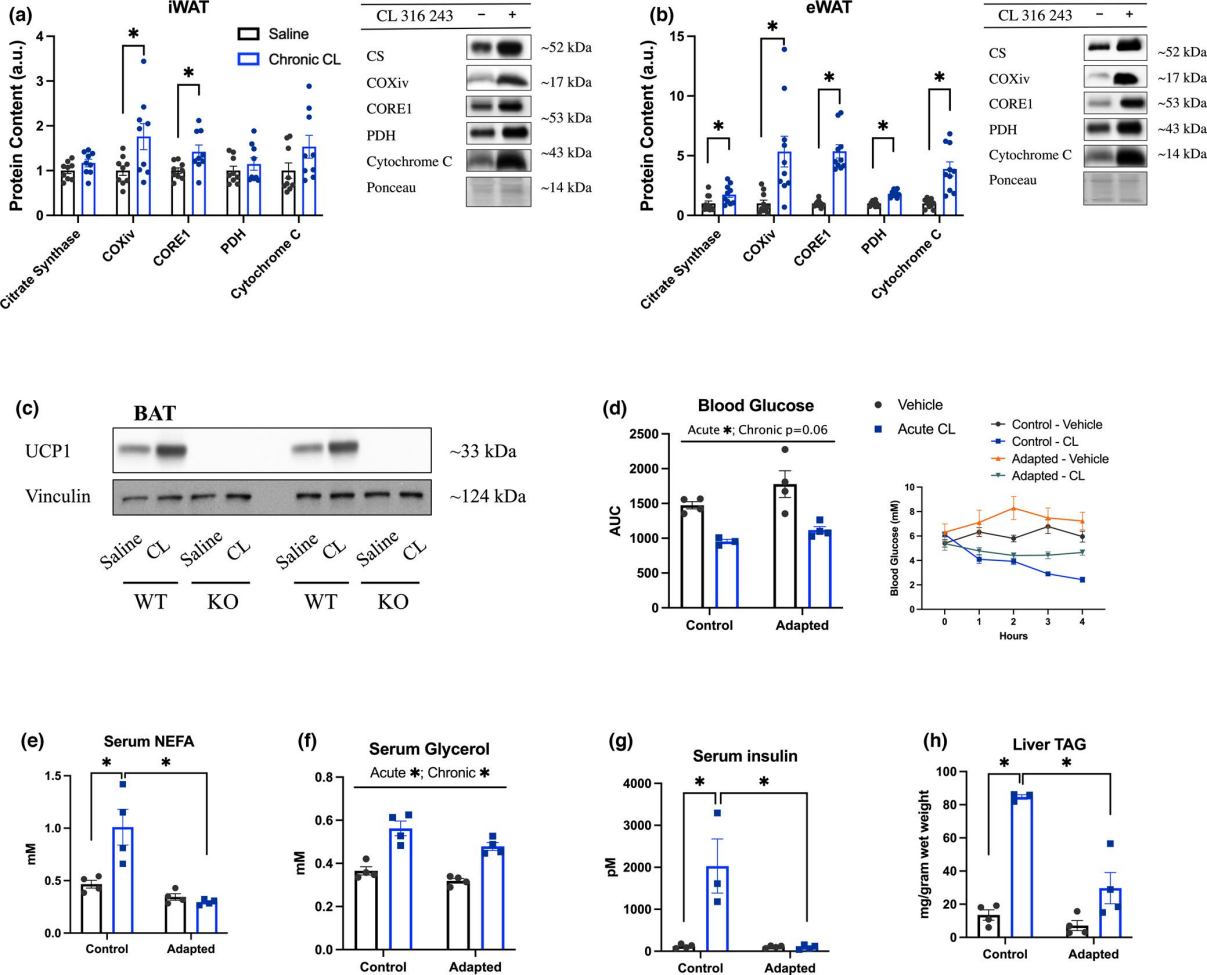
In UCP1 knockout mice the chronic effects of CL to induce mitochondrial biogenesis are intact and the acute metabolic effects of CL dissipate with repeated use. UCP1^−/−^ mice were adapted to CL or saline injections for 6 days. On the seventh day (24 h following the last treatment) adipose depots were dissected and analyzed for protein content via western blot (a–c; *n* = 5–6 mice/group). Proteins of interest were expressed relative to an internal loading control from the same respective gel as the protein of interest. Please note that a ponceau stain was used for correction purposes for each protein of interest but for space purposes only one representative ponceau is shown for each tissue. UCP1 protein was undetectable in brown adipose (BAT) from treated and untreated knockout mice. In a separate cohort, mice were treated with CL or saline for 6 days then on the seventh day were treated acutely with CL or saline. Blood glucose (d; *n* = 3–4 mice/group) was measured each hour for 4 h for determination of AUC (glucose curve inset). Four hours following acute treatment serum, liver, and WAT depots were collected for analysis of serum NEFA (e; *n* = 4 mice/group), glycerol (f; *n* = 4 mice/group), insulin (g; *n* = 3–4 mice/group), and liver TAG accumulation (h; *n* = 3–4 mice/group). Protein content (a, b) was analyzed by two‐tailed unpaired *t*‐test while differences between groups in (d–h) were analyzed by two‐way ANOVA. *p < 0.05 between indicated groups. All data are presented as mean ± SEM

It has been proposed that compensatory increases in insulin, caused by the mobilization of lipids, may be necessary for the provision of substrates to fuel active brown adipose tissue (Heine et al., 2018). We thus aimed to determine if acute CL‐induced increases in lipid mobilization and WAT adaptations with repeated CL treatment are present in mice with reductions in brown adipose activity. To address this question, we completed our 6‐day CL or saline treatment protocol (1 mg/kg) in UCP1^−/−^ mice, a model in which CL‐induced increases in energy expenditure and BAT thermogenesis are absent (Atgié et al., 1998; Granneman et al., 2003). Similar to what was reported in our experiments with C57BL6J mice, repeated treatment with CL increased the protein content of mitochondrial proteins in both iWAT (COXIV, CORE1) (Figure 4a) and eWAT (citrate synthase, COXIV, CORE1, PDH, Cytochrome C) (Figure 4b). As expected, UCP1 protein content was absent in BAT from UCP1^−/−^ mice (Figure 4c). On the seventh day of treatment half of the animals of each group were treated acutely with CL (1 mg/kg) or saline. Blood glucose was measured over 4 h and revealed a main effect of acute CL to reduce blood glucose AUC and a trend (*p* = 0.06) for chronic CL treatment to increase blood glucose AUC (Figure 4d). Serum NEFA measured after 4 h of treatment was increased in the control‐acute CL group but not in the adapted‐acute CL group (Figure 4e). Serum glycerol displayed main effects to be increased by acute CL and reduced by chronic adaptation to CL (Figure 4f). Serum insulin (Figure 4g) and liver TAG accumulation (Figure 4h) were significantly increased in the control‐acute CL group only. While it has been suggested that the CL‐induced increases in insulin due to lipid mobilization is an adaptive response for the provision of substrates to activated brown adipose tissue (Heine et al., 2018; MacPherson et al., 2014); we provide evidence to the contrary, as lipid mobilization is intact in UCP1^−/−^ mice, a phenotype that has reduced CL‐induced BAT activity (Granneman et al., 2003).

### 3.5 The abrogated acute effects of CL caused by repeated treatment are rescued by co‐treatment with cilostamide

**FIGURE 5:**
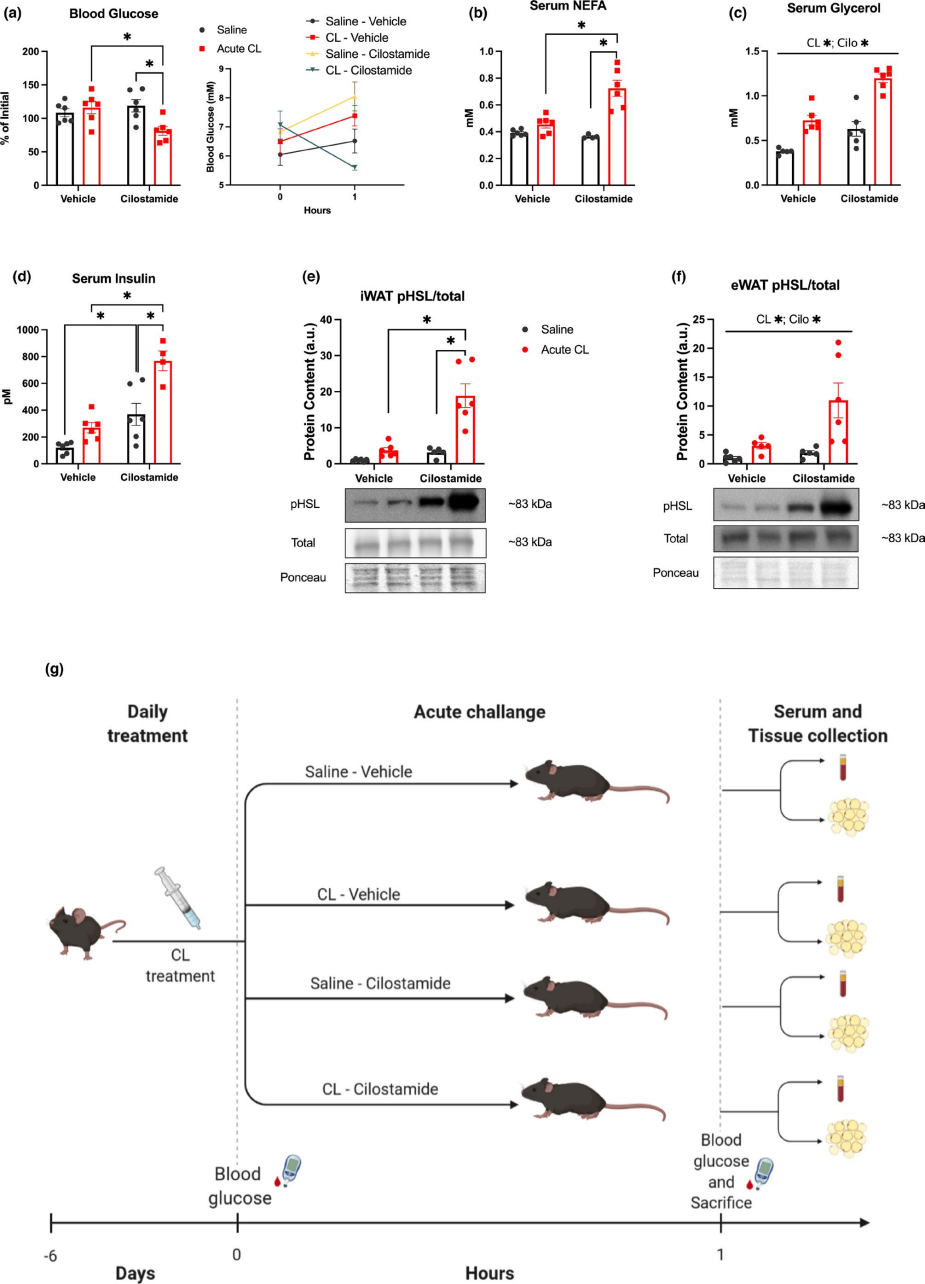
The acute effects of CL are rescued by co‐treatment with cilostamide. Mice were adapted to CL for 6 days then treated acutely with CL and or cilostamide. (a; *n* = 6 mice/group) Blood glucose was measured prior to acute injection and after 1 h and displayed as a percentage of initial (absolute blood glucose in mM inset). One hour following acute treatment serum and WAT depots were collected for analysis of serum NEFA (b; *n* = 4–6 mice/group), glycerol (c; *n* = 5–6 mice/group), insulin (d; *n* = 4–6 mice/group), and WAT HSL phosphorylation (serine 660) via western blot in iWAT (e) and eWAT (f) depots. Proteins of interest were expressed relative to an internal ponceau loading control from the same respective gel as the protein of interest. Please note that a ponceau stain was used for correction purposes for each protein of interest but for space purposes only one representative ponceau is shown for each tissue. Phosphorylation of proteins are represented relative to their respective total protein content and ponceau stain loading control (representative images inset). (g) Schematic representation of the study design. All mice within this cohort were given repeated treatment with CL, then split into acute treatment groups where half were treated acutely with CL and/or cilostamide. Differences between groups were analyzed by two‐way ANOVA. *p < 0.05 between indicated groups. All data are presented as mean ± SEM

The current data provides evidence of a desensitization to the acute metabolic effects of CL, an effect observed with other interventions which activate β3ARs such as chronic cold stress. Cold stress leads to increases in phosphodiesterase activity (Heine et al., 2018), a class of enzymes that hydrolyze cAMP and subsequently reduce PKA activity, and since we saw reductions in CL‐induced increases in HSL phosphorylation on a PKA phosphorylation site, we aimed to assess the efficacy of the phosphodiesterase 3 inhibitor cilostamide to rescue the desensitization caused by repeated CL treatment on WAT. To this end we treated all mice with CL (1 mg/kg) for six consecutive days and on the last day of treatment assessed acute metabolic effects in these CL‐adapted mice by treating half of the mice with saline and the remainder with CL. Half of each acute treatment group (saline or CL) was co‐treated with cilostamide. As in our previous experiments, acute CL‐mediated reductions in blood glucose (Figure 5a) and increases in serum NEFA (Figure 5b) and insulin (Figure 5d) were absent in mice that had been repeatedly treated with CL and this was rescued by co‐treatment with cilostamide. Similarly, we found main effects of acute CL and co‐treatment with cilostamide to increase serum glycerol (Figure 5c).

The inhibition of phosphodiesterase should reduce the hydrolysis of cAMP and therefore increase the activation of PKA upon stimulation of the beta‐adrenergic signaling pathway. Consistent with this notion we found that acute CL‐induced increases in HSL phosphorylation were increased in iWAT from mice co‐treated with cilostamide while there were main effects of cilostamide and CL to increase HSL phosphorylation in eWAT. These findings, together with our glucose, insulin, and NEFA data, provide evidence that the desensitization to repeated CL treatment can be rescued by cilostamide.

## 4 DISCUSSION

Repeated activation of β3ARs results in remodeling of white adipose tissue characterized by increased mitochondrial enzymes, the expression of UCP1, and the appearance of multilocular fat cells (Buzelle et al., 2015; Mottillo et al., 2014, 2016). These features occur most prominently in iWAT and are paralleled by increases in energy expenditure and reductions in fat mass (Buzelle et al., 2015; Mottillo et al., 2014). An underappreciated aspect of β3AR activation is the rapid and robust effects it has on lowering blood glucose. The glucose lowering effects of β3AR agonists such as CL are thought to be due to fatty acid‐mediated increases in circulating insulin concentrations (MacPherson et al., 2014, 2016). In support of this supposition, we have previously shown that the genetic deletion of the lipolytic enzyme adipose triglyceride lipase (ATGL) or the pharmacological inhibition of lipolysis with nicotinic acid attenuates CL‐induced increases in insulin and reductions in blood glucose (MacPherson et al., 2014, 2016). In the current paper we demonstrate that the glucose lowering effects of CL are dampened with repeated dosing and similar to our previous work is associated with reductions in CL‐induced increases in markers of lipolysis, that is, adipose tissue HSL phosphorylation and serum NEFA, and circulating insulin concentrations. Importantly we were able to demonstrate attenuated responses to CL across several different models (low‐fat diet, high‐fat diet, and UCP1^−/−^ mice) at different time points, and both in vivo and in tissue explants. These findings are consistent with previous work demonstrating a desensitization to adrenergic stimuli in rodent eWAT and brown adipose following chronic cold exposure or treatment with beta adrenergic agonists (Atgié et al., 1998; Unelius et al., 1993).

While we demonstrate that the repeated activation of the β3AR results in a desensitization to the acute glucose lowering effects of CL, it should be noted that the ability of CL to increase energy expenditure remained intact, and if anything, increased in mice that had been repeatedly treated with CL before the acute measurement of substrate oxidation. This finding is consistent with previous work from Granneman et al. in mice housed at room temperature and repeatedly dosed with CL (Granneman et al., 2003). While Heine et al. (2018) have pointed to the possibility that CL‐induced increases in insulin (MacPherson et al., 2014) serves to provide substrates to activated brown adipose tissue, several pieces of data in the current investigation would argue against this. First, we show that acute CL‐induced increased in energy expenditure are maintained in mice habituated to CL treatment, a condition in which CL‐induced increases in serum NEFA and insulin are attenuated. Second, and perhaps more importantly, CL‐mediated increases in lipid mobilization and insulin are intact in drug naïve UCP1^−/−^ mice, a model that displays markedly reduced BAT activity with CL stimulation at thermal neutrality (Crane et al., 2014). While speculative, perhaps the increase in insulin with CL treatment serves as a feedback mechanism to limit fatty acid release, rather than to provide substrate to brown adipose tissue.

The mechanisms which could be underlying reductions in beta adrenergic responsiveness following repeated CL treatment and which could be potentially targeted to alleviate this, have not been elucidated, but would not appear to be explained by alterations in the expression of the β3AR itself, as we did not detect changes in the expression of this receptor, at least at the mRNA level. Prior work utilizing brown adipocytes from cold acclimated hamsters has reported increases in phosphodiesterase activity (Unelius et al., 1993), an enzyme which hydrolyzes cAMP, and which would be expected to subsequently reduce the activation of PKA and the phosphorylation of its downstream targets. Consistent with this notion we found that CL‐mediated increases in the phosphorylation of HSL on serine 660, a PKA phosphorylation site, and the induction of PKA‐ targeted genes such as UCP1 and PGC1α were absent in iWAT and eWAT from mice repeatedly treated with CL. Given these findings we hypothesized that the pharmacological targeting of phosphodiesterase would be sufficient to overcome desensitization to CL. To accomplish this, we co‐treated mice with the phosphodiesterase inhibitor cilostamide. This drug, or similar analogues have been used clinically as a vasodilator and antiplatelet therapy (Hidaka et al., 1979; Sudo et al., 2000). In mice which had been adapted to repeated CL treatment, co‐treatment with cilostamide resulted in increases in HSL phosphorylation, serum NEFA, and insulin and reductions in blood glucose in comparison to mice that did not receive the cilostamide co‐treatment.

Several limitations to the current study need to be recognized. First, while we found that pharmacological targeting of phosphodiesterase was sufficient to rescue desensitization to CL, this was only tested in lean animals and it is yet to be determined if a similar effect is seen in other models, that is, high‐fat fed and/or UCP1^−/−^ mice. Second, it remains unclear if repeated targeting of phosphodiesterase would result in desensitization to the effects of cilostamide, though it should be noted that this has not been reported in mice treated with six doses over 3 days (Guirguis et al., 2013). Lastly, given differences in beta adrenergic receptor expression between rodents and humans (Ito et al., 1998), and potential unwanted side effects of cilostamide such as tachycardia (Kaumann et al., 2009) future investigations are required to determine if co‐treatment with beta adrenergic agonists and phosphodiesterase inhibitors would be an efficacious approach to improve glucose metabolism in humans.

**FIGURE 6:**
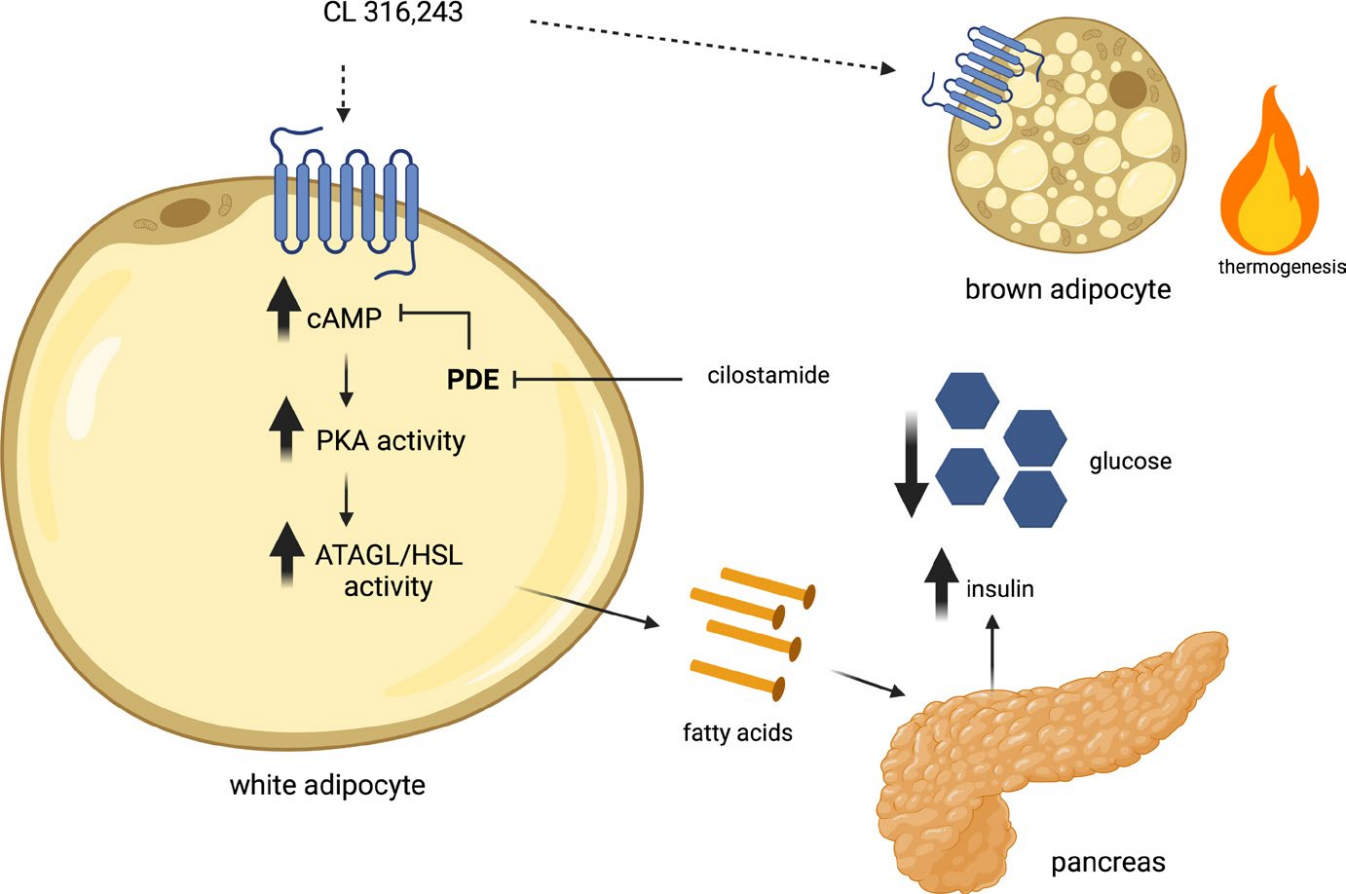
A proposed model of the interactions between treatment with CL 316,243, lipolysis, insulin release, and energy expenditure

In summary we have demonstrated that repeated treatment with the β3AR agonist CL, leads to an attenuated ability of this compound to reduce blood glucose, likely secondary to reductions in lipolysis and insulin secretion, an effect that can be rescued through the pharmacological inhibition of phosphodiesterase with cilostamide (Figure 6). Results from the current investigation also highlight an uncoupling between CL‐induced increases in serum insulin levels and subsequent reductions in blood glucose from energy expenditure/brown adipose tissue thermogenesis. The current findings not only increase our fundamental understanding of how adipose tissue regulates integrative metabolism, but also provides important insights into co‐treatment strategies that could be used to regulate glucose metabolism.

## CONFLICT OF INTEREST

There are no conflict of interest to declare.

## AUTHOR CONTRIBUTIONS

KDM and DCW conceived and designed experiments. KDM, GLM, and HS conducted the experiments. KDM, GLM, HS, IS, and DCW analyzed data and prepared figures. KDM and DCW drafted the manuscript. All authors edited and approved the final version of the manuscript. All those who qualify for authorship are listed.

## ETHICS STATEMENT

All experimental procedures were approved by the University of Guelph Animal Care Committee and followed Canadian Council on Animal Care guidelines.

## Supporting information



Figure S1

Table S1

Table S2
