# Supplementary material for: The glucose lowering effects of CL 316,243 dissipate with repeated use and are rescued bycilostamide
Source: Physiol Rep. 2022 Feb 18;10(4):e15187. doi: 10.14814/phy2.15187 (PMC8855634; doi:10.14814/phy2.15187)
Supplement: Supplementary file 1 — Figure S1 [file PHY2-10-e15187-s001.pdf]

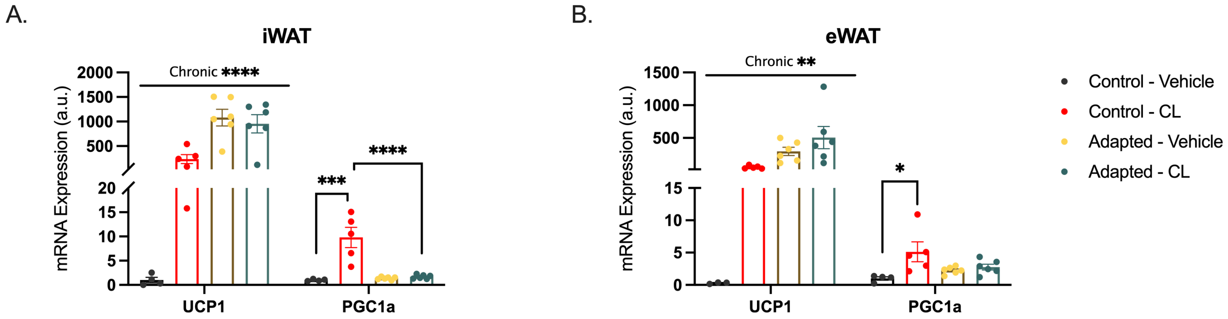

Supplemental Figure 1: Relative mRNA expression measured in iWAT and eWAT depots after 1 hour of acute CL treatment. Mice were treated with CL or saline for 6 days then on the seventh day were treated acutely with CL or saline (n=3-6 mice/group). One hour after acute treatment iWAT (**A**) and eWAT (**B**) depots were collected for determination of mRNA expression of UCP1 and PGC1a. Differences between groups were analyzed by two-way ANOVA. *P < 0.05, **P < 0.01, ***P < 0.001, ****p<0.0001 between indicated groups. All data are presented as mean ± SEM.
