# Supplementary material for: The glucose lowering effects of CL 316,243 dissipate with repeated use and are rescued bycilostamide
Source: Physiol Rep. 2022 Feb 18;10(4):e15187. doi: 10.14814/phy2.15187 (PMC8855634; doi:10.14814/phy2.15187)
Supplement: Supplementary file 2 — Table S1 [file PHY2-10-e15187-s002.pdf]

**Supplemental Table 1:** mRNA primer sequences used.			
Gene	Forward Sequence	Reverse Sequence	Reference
Ppib	*GGAGATGGCACAGGAGGAA*	*GCCCGTAGTGCTTCAGCTT*	[28]
Ucp1	*ACTGCCACACCTCCAGTCATT*	*CTTTGCCTCACTCAGGATTGG*	[29]
Pgc1a	*CCCTGCCATTGTTAAGACC*	*TGCTGCTGTTCCTGTTTTC*	[29]
β3AR	*GGCCCTCTCTAGTTCCCAG*	*TAGCCATCAAACCTGTTGAGC*	[30]
