# Supplementary material for: The glucose lowering effects of CL 316,243 dissipate with repeated use and are rescued bycilostamide
Source: Physiol Rep. 2022 Feb 18;10(4):e15187. doi: 10.14814/phy2.15187 (PMC8855634; doi:10.14814/phy2.15187)
Supplement: Supplementary file 3 — Table S2 [file PHY2-10-e15187-s003.pdf]

SUPPLEMENTAL TABLE 2: SERUM METABOLITES 1-HOUR POST-TREATMENT

	**Treatment Groups**				**P Value**		
	**Control-Vehicle**	**Control-CL**	**Adapted-Vehicle**	**Adapted-CL**	**Acute CL**	**Chronic**	**Acute × Chronic**
**NEFA** (mM)	0.72 ± 0.04[a]	1.27 ± 0.11[b]	0.65 ± 0.16[a]	0.58 ± 0.03[a]	0.0189	0.0007	0.0043
**GLYCEROL** (mM)	0.52 ± 0.05	0.86 ± 0.06	0.53 ± 0.09	0.65 ± 0.02	0.0006	0.0849	0.0650
**INSULIN** (pM)	119.51 ± 24.82[a]	1252.66 ± 455.42[b]	432.66 ± 205.52[a]	192.44 ± 40.75[a]	<0.0001	<0.0001	<0.0001

Notes: Data are represented as the mean ± SEM. NEFA, non-esterified fatty acids; Acute CL, P value for main effect of acute CL 316,243 treatment; Chronic, P value for main effect of adaptation to 6 days of CL 316,243 treatment; Acute × Chronic, P value for interaction between acute CL treatment and adaptation to chronic CL treatment (NEFA and Glycerol n=6-8 per group; Insulin n=3-8 per group). Different superscript lowercase letters denote a significant post hoc comparison.
